# *Porphyromonas gingivalis* and its lipopolysaccharide differently modulate epidermal growth factor–dependent signaling in human gingival epithelial cells

**DOI:** 10.1080/20002297.2017.1334503

**Published:** 2017-06-15

**Authors:** R. Elkaim, I. M. Bugueno-Valdebenito, N. Benkirane-Jessel, H. Tenenbaum

**Affiliations:** ^a^ University of Strasbourg, Dental Faculty, Strasbourg, France; ^b^ INSERM 1109 ‘Osteoarticular and Dental Regenerative Nanomedicine’, Fédération de Médecine Translationnelle de Strasbourg (FMTS), Strasbourg, France

**Keywords:** EGF – signaling, *Porphyromonas gingivalis*, human gingival epithelial cells

## Abstract

Periodontitis is an inflammatory disease induced by pathogenic bacteria such as *Porphyromonas gingivalis*. Little is known about epidermal growth factor (EGF) signals in human gingival epithelial cells (HGEC), which are major targets of *P. gingivalis*, and how the expression of proteins participating in EGF signaling—that is, EGF-receptor (EGFR), suppressor of cytokine signaling-3 (SOCS-3), interferon regulatory factor-1 (IRF-1), and signal transducers and activators of transcription (STAT-3)—are modified. This study aimed to assess the effects of *P. gingivalis* and its purified lipopolysaccharide (LPS-*Pg*) on EGF signaling. HGEC were infected for 2 h in a dose-dependent manner with *P. gingivalis* and with heat-killed *P. gingivalis*, and activated for 2 and 24 h by 1 µg/mL of purified LPS-*Pg*. Quantitative reverse transcription polymerase chain reaction and Western blotting were performed to measure mRNA and protein levels for SOCS-3, IRF-1 EGF, EGFR, and STAT-3. The tyrosine-phosphorylation status of STAT-3 was also examined. The results showed that infection of HGEC cells with *P. gingivalis*, but not with heat-killed *P. gingivalis*, led to significant reductions in expression levels of mRNAs and proteins for SOCS-3, IRF-1, and EGFR, while LPS-*Pg* over time significantly increased the expression of these mRNAs and proteins. Tyrosine-phosphorylation of STAT-3 was significantly increased during infection with *P. gingivalis* and activation by LPS-*Pg* but not modified during infection with heat-killed *P. gingivalis*. This study highlights that *P. gingivalis* and its purified LPS differentially modulated the expression of proteins (SOCS-3, IRF-1, EGFR, and STAT-3) interfering with EGF signaling.

## Introduction

Periodontal diseases are inflammatory diseases affecting tooth-supporting tissues and are induced by oral bacterial biofilms composed of >500 species. In addition to a notably impaired oral status, the negative influence of periodontal diseases on general health is revealed in cases of severe periodontitis [1]. One of the most pathogenic bacteria is *Porphyromonas gingivalis*, a Gram-negative anaerobe bacterium that spreads from periodontal pockets to the general circulation and has been associated with systemic diseases such as rheumatoid arthritis, atherosclerosis, and diabetes [2,3]. The local host response to these bacteria includes activation of resident cells (epithelial cells, fibroblasts, endothelial cells, and macrophages), recruitment of inflammatory cell types (monocytes, lymphocytes, and neutrophils), and the subsequent release of inflammatory mediators and cytokines (interleukin [IL]-1, IL-6, IL-8, and tumor necrosis factor alpha), which play crucial roles in periodontal tissue destruction. Among these cell-derived factors are members of the epidermal growth factor (EGF) family, potential ligands for the epidermal growth factor receptor (EGFR). EGF was originally described as acting in a paracrine fashion on keratinocytes, and *in vitro* studies have shown that EGF is upregulated after acute injury, significantly accelerating re-epithelialization and increasing tensile strength in wounds [4]. EGF is also involved in a large array of cellular functions such as periodontal regeneration, cell motility, migration, and differentiation [5–7]. EGF and its receptor are also implicated in periodontitis, but little is known about the regulation of the EGF pathway during periodontitis [8,9]. Significant differences were found in EGF levels between healthy and diseased subjects [10]. Patients with periodontal pockets <5 mm have a reduced EGF concentration compared to patients with periodontal pockets >5 mm. Additionally a single nucleotide mutation within the EGF coding sequence was associated with the development of severe chronic periodontitis [11]. Concerning EGFR, its expression seemed to be limited to the gingival epithelium, since a very low level of expression appeared within the periodontal ligament. However, within inflamed gingiva and within tissues observed after guided tissue regeneration (GTR), reports indicated a considerable increase [12,13]. Therefore, the balance between EGF and EGFR could constitute an important mechanism of regulation during the pathogenicity process of periodontal diseases, as observed with enzymes such as cathepsins and matrix metalloproteinases with their natural inhibitor counterparts [14].

EGF is a small protein expressed as a pro-form that is proteolytically processed into an active peptide encompassing 33 amino acid residues. The binding of the active peptide to EGFR leads to the subsequent activation of the receptor through dimerization, which requires intrinsic tyrosine-kinase activity [15]. Once activated, the EGFR initiates downstream signaling cascades such as the Tyr^705^ phosphorylation of the transcription factor STAT-3 (signal transducers and activators of transcription) initiated by JAK (Janus kinase), leading to its translocation within the nucleus with consequences for the activation and repression of genes that facilitate proliferation, regeneration, and tumor genesis [16].

In periodontitis, many virulence factors that originate from *P. gingivalis* (i.e. gingipains, fimbriae, and lipopolysaccharide [LPS]) may disturb the EGF signaling pathway. Indeed, in a model of gingival fibroblasts, purified LPS from *P. gingivalis* led to a loss of activation of secondary signals depending on the presence of EGF, notably ERK1/2 and p38 kinase, with downstream consequences for the phosphorylation of the transcription factor cAMP response element-binding [17]. In addition to LPS and other known virulence factors, one bacterial enzyme, peptidyl arginine deiminase (PADPg), a unique feature from *P. gingivalis*, is emerging [18]. This bacterial enzyme can citrullinate polypeptides but in a different way from its five human counterparts [19]. PADPg may play a role in EGF signaling, as suggested by an *in vitro* study showing that EGF can be a substrate of the purified PADPg [20]. Citrullination of the C-terminal arginine of purified EGF by PADPg distorted the cross-talk between the epithelium and EGF, thus hindering its ability to stimulate epidermal cell proliferation. Citrullination by PADPg may also concern a variety of other peptides, including the vasoregulatory peptide hormone bradykinin [21]. In addition to EGF, the bacterial PAD abrogated the mRNA expression of two genes known to be implicated in the EGF-mediated effect, the suppressor of cytokine signaling-3 (SOCS-3), a STAT-regulated cytokine-inducible negative regulator of cytokine signaling, and the interferon regulatory factor-1 (IRF-1), an activator of alpha- and beta-interferon transcription [22]. SOCS proteins are negative feedback regulators of cytokine signaling mediated by the JAK-STAT signaling pathway, thus playing pivotal roles in various physiological processes such as inflammation, including bone inflammatory responses, and the development and progression of cancers [23,24]. IRF-1 belongs to a family of transcription factors that regulate cellular responses by targeting genes induced by interferon gamma (INF-γ), such as IP-10 (INF-γ-inducible protein-10), ITAC (interferon-inducible T-cell alpha chemo attractant), or Mig (monokine induced by INF-γ) [25]. Whether the *in vitro* effects observed with purified bacterial PAD on EGF-signaling expression occurred physiologically in tissue damage and delayed healing within *P. gingivalis*–infected periodontium remains unknown. Therefore, this study explored the effect of *P. gingivalis* and its purified LPS on the mRNA and protein expressions of molecules involved in the EGF signaling pathway, notably SOCS-3, IRF-1, EGFR, and STAT-3, with possible consequences on the activation of STAT-3 by tyrosine phosphorylation in human gingival epithelial cells (HEGC).

## Materials and methods

### Bacterial culture

*P. gingivalis* strain (ATCC 33277) was purchased from the American Type Culture Collection (ATCC, Manassas, VA). Bacterial culture was performed under strict anaerobic conditions at 37°C in brain heart infusion (BHI) medium supplemented with menadione (1 μg/mL) and hemin (5 µg/mL), both purchased from Sigma–Aldrich (St. Louis, MO). The day of infection, bacteria were pelleted by centrifugation, washed twice with phosphate-buffered saline (PBS), and suspended in 1 mL of sterile PBS. The concentration of bacteria was determined optically at OD_600_ (1 OD = 5 × 10^8^ bacteria/mL), as previously described [26]. *P. gingivalis* was also heat killed at 85°C for 10 min before the experiment. Bacterial inactivation was confirmed by determination of the absence of colony-forming units (CU/mL) after anaerobic cultivation.

Commercial ultrapure LPS-*Pg* was purchased from InvivoGen (San Diego, CA), dissolved in ultra-pure water at a final concentration of 1 mg/mL, and conserved at −80°C by aliquots.

### Cell culture

HGEC were cultured in a ready-to-use Keratinocytes-SFM medium (Life Technologies, Carlsbad, CA) supplemented with human recombinant epidermal growth factor (rEGF) and bovine pituitary extract, and antibiotics (penicillin: 100 IU/mL; streptomycin: 100 mg/mL; total medium). Cells were cultured at 37°C in a humidified atmosphere with 5% CO_2_. All the experiments were performed with cells between passages 3 and 5.

### Infection of HGEC with *P. gingivalis*

The day before the experimentation, 2 mL of HGEC at 5 × 10^4^ cells/mL (for extraction of total RNA) and at 2.5 × 10^6^ cells/mL (for preparation of cellular or nuclear extracts) were plated on culture dishes (5 cm diameter) in the total medium but without antibiotics. On the day of infection, cells were washed twice with PBS and infected for 2 h with *P. gingivalis* or heat-killed *P. gingivalis* at a multiplicity of infection (MOI) of 10 and 100 bacteria per cell in the total medium without antibiotics. Uninfected HGEC cells served as controls. At the end of the infection, HGEC were washed at least twice with cold-sterile PBS to eliminate the bacteria completely. Total RNA and cellular and nuclear extracts were then prepared.

#### Activation of HGEC by LPS-Pg purified from *P. gingivalis*

A total of 2 mL of HGEC at 5 × 10^4^ cells/mL (for extraction of total RNA) and at 2.5 × 10^6^ cells/mL (for preparation of cellular or nuclear extracts) were plated on culture dishes (5 cm diameter) in the total culture medium. The next day, the medium was changed. The cells were then washed twice with PBS and activated for 2 and 24 h by adding 1 µg/mL of purified LPS-*Pg* in the total HGEC culture medium. Plates without LPS-*Pg* served as controls. At the end of the infection, HGEC were washed at least twice with cold-sterile PBS. Total RNA and cellular and nuclear extracts were then prepared.

#### Quantitative reverse transcription polymerase chain reaction

Total RNA was isolated using the High Pure RNA Isolation kit (Roche Applied Science, Meylan, France) according to the manufacturer’s instructions. RNA quality and concentration were determined spectrophotometrically using a NanoDrop 1000 apparatus (Fisher Scientific, Illkirch, France). Total RNA (1 µg) was used as a template for cDNA synthesis using the iScript Reverse Transcriptase Supermix (Bio-Rad Laboratories, Hercules, CA) in conditions described by the furnisher. A negative control was performed using total RNA without enzyme. Primers were designed against the following genes: β-actin, EGF, and its receptor EGFR, SOCS-3, IRF-1, STAT-3, and PADPg. Validated primers (Quantitect primers) for all genes were obtained from Qiagen France (Courtaboeuf, France) except for the bacterial PAD (forward primer: 5ʹ-CCT TGG GGG TAC TGC ATC GTG-3ʹ; reverse primer: 5ʹ-ACC TCG ATG CCG TCG CC CTC TCG-3ʹ) and STAT-3 (forward primer: 5’-ATC TCC AGG ATG ACT TTG AT-3ʹ; reverse primer: 5ʹ-AGT TTT CTG CAC ATA CTC CA-3ʹ) that were all purchased from Thermo Fisher Scientific (Saint-Aubin, France).

For quantitative reverse transcription polymerase chain reaction (RT-qPCR), equal amounts of cDNA (200 ng) were used with the iTaq^Tm^ Universal SyB®Green Supermix (Bio-Rad Laboratories) with primers concentrations and conditions indicated by the furnisher. A Bio-Rad iCycler IQ with iCycler IQ software v3.1 (Bio-Rad Laboratories) was used with the auto-calculated threshold cycle (C_T_) selected. β-Actin was selected as stable reference gene under the experimental conditions of this study. The cycle threshold (C_T_) value of each gene was normalized to β-actin using the comparative 2^–^^∆∆CT^ method.

#### Preparation of cellular extracts

Cell culture media was removed, and infected or activated cells were washed twice with ice-cold PBS. Cells were collected by scraping into 5 mL of ice-cold PBS and harvested by centrifugation at 1000 *g* for 5 min at 4°C. The medium was completely aspirated, and cells were lysed for 10 min at 4°C by the addition of 200 µL of the extraction buffer (50 mM of HEPES pH 7.0, 250 mM of NaCl, 5 mM of EDTA, 0.1% Nonidet P-40, 50 mM of NaF, 0.5 mM of Na_3_VO4, 1 mM of phenylmethylsulphonyl fluoride (PMSF) containing 5 μg/mL each of aprotinin, leupeptin, and pepstatin, all compounds from Sigma–Aldrich). Cell debris was removed by centrifugation at 1000 *g* followed by quick freezing of the supernatant. The protein concentration in the supernatant was determined using the Bio-Rad DC assay (Bio-Rad Laboratories), and 5 µg of protein was immediately used for Western blotting.

### Extractin of nuclear extracts

Infected and activated HGEC were washed twice with ice-cold PBS, scraped into 5 mL of cold PBS, and collected by centrifugation. Cell pellets were suspended in hypotonic buffer (10 mM of HEPES, pH 7.9, 1.5 mM of MgCl_2_, 10 mM of KCl, 0.2 mM of PMSF, 0.5 mM of DTT, 10 μg/mL of aprotinin) and incubated on ice for 15 min. Cells were then lysed by adding 0.1% Nonidet P-40 and vortexed vigorously for 10 s. Nuclei were pelleted by centrifugation at 12,000 *g* for 1 min at 4°C and suspended in 200 µL of high salt buffer (20 mM of HEPES, pH 7.9, 25% glycerol, 400 mM of KCl, 1.5 mM of MgCl_2_, 0.2 mM of EDTA, 0.5 mM of DTT, 1 mM of NaF, 1 mM of Na_3_VO4). The protein concentration was determined with the Bio-Rad DC assay (Bio-Rad Laboratories), and 20 µg of protein was immediately used for Western blotting to visualize the phosphorylated form of STAT-3 (p-STAT-3).

### Western immunoblotting

Cellular or nuclear extracts were used for SDS-PAGE electrophoresis, as previously described [26]. Proteins were transferred by electroblotting onto nitrocellulose membranes, and blots were blocked for 2 h at room temperature in Tris-buffered saline (TBS) containing either 5% skim dry milk or 5% bovine serum albumin, depending on the primary antibody used. Except for the anti-EGF (cat: WHOOO1950M1), the anti-STAT-3 (cat: 07–2173), and the anti-phospho-STAT-3 (pTyr^705^; cat: SAB4300033), which were obtained from Sigma–Aldrich, all other primary and secondary antibodies were purchased from Proteintech Europe (Manchester, United Kingdom) and were used at the concentrations recommended by the furnisher: anti-IRF-1 polyclonal antibody (cat: 11335–1-AP); anti-SOCS-3 polyclonal antibody (cat: 14025–1-AP); anti-EGFR (cat: 18986–1-AP); and anti-GAPDH polyclonal antibody (cat: 10494–1-AP). Primary antibodies were incubated overnight at 4°C under gentle agitation. Antigen–antibody binding was detected using horseradish peroxidase conjugated species-specific secondary antibody for 2 h at room temperature followed by several washes with TBS containing 0.05% Tween 20. Enhanced chemiluminescence substrates for horseradish peroxidase (Perkin-Elmer, Courtaboeuf, France) were applied and signals acquired and quantified using the Image Lab Software v3.0 (Bio-Rad Laboratories). The intensity determined for a specific band in an assay was normalized to the intensity of the internal control GAPDH band obtained within the same assay.

To measure the phosphorylation level of STAT-3, the band intensity obtained with the anti-phospho-STAT-3 (Tyr^705^) antibody in an assay was expressed relative to the intensity of the band obtained with the antibody detecting the total antigen STAT-3 in the same assay.

### Statistical analysis

All experiments were repeated at least three times, and the results are expressed as the mean ± standard deviation. To determine the significance of the obtained results, comparisons between groups were made using Student’s *t*-test. *p*-Values of <0.05 were considered significant.

## Results

### P. gingivalis inhibited the mRNA expression of SOCS-3, IRF-1, EGF, and EGFR but not of STAT-3

**Figure 1.**
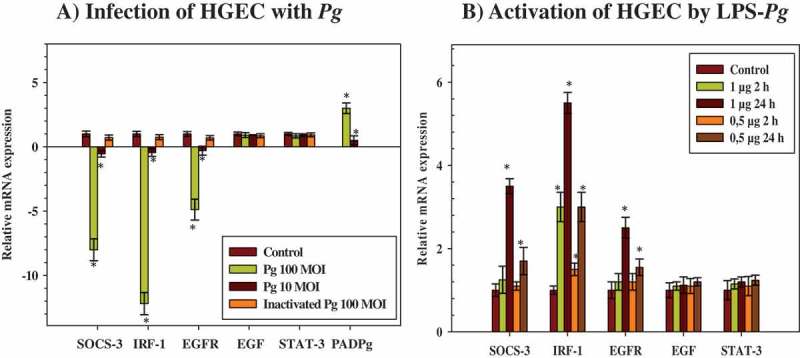
Modulation of the expression of microRNAs (mRNAs) encoding cytokine signaling-3 (SOCS-3), interferon regulatory factor-1 (IRF-1), epidermal growth factor (EGF), EGF-receptor (EGFR), and signal transducers and activators of transcription (STAT-3) in human gingival epithelial cells (HGEC) infected with *Porphyromonas gingivalis* and activated by its lipopolysaccharide (LPS-*Pg*). (a) HGEC were infected for 2 h with *P. gingivalis* at different multiplicities of infection (MOI; 10 or 100) and with heat-killed *P. gingivalis* (Ina) at a MOI of 100. Uninfected cells served as control. (b) HGEC were activated for 2 h and 24 h by 0.5 µg/mL and 1 µg/mL of LPS-*Pg*. Unactivated cells served as controls. In both experiments, total RNA were prepared, and levels of mRNAs encoding SOCS-3, IRF-1, EGF, EGFR, and STAT-3 were determined by quantitative reverse transcription polymerase chain reaction analysis. All results were presented as the quantity relative to β-actin as a reference gene. Differences (*) between a test mRNA and the control HGEC were analyzed with Student’s *t*-test (*p* < 0.0005). All experiments were repeated three times, and the results are expressed as mean ± the standard deviation.

The study sought to establish whether the infection of HGEC with live *P. gingivalis* would affect the mRNA expression of various genes potentially implicated in EGF signaling, mainly SOCS-3, IRF-1, EGF, EFGR, and STAT-3. HGEC were infected for 2 h with live and heat-killed *P. gingivalis* at a MOI of 100. Total RNA was extracted, and the mRNA expression levels of the tested genes were measured by RT-qPCR. The mean data obtained from three different experiments showed that the levels of expression of the mRNAs encoding for SOCS-3, IRF-1, and EFGR were significantly downregulated in infected HGEC (by 8-, 12-, and 5-fold, respectively) compared to corresponding levels measured in uninfected control cells (Figure 1a). These decreases were *P. gingivalis* dose-dependent, since an infection with *P. gingivalis* at a MOI of 10 considerably attenuated these effects (by twofold for each mRNA when compared to controls). The expression levels of mRNAs encoding EGF and STAT-3 did not vary at any MOI used, but it is notable that the expression of the mRNA encoding EGF was very low and even undetectable in some experiments. The infection of HGEC performed with heat-killed *P. gingivalis* at a MOI of 100 did not significantly modify the levels of mRNAs encoding for all the tested genes, suggesting that the downregulated effects observed with live *P. gingivalis* might originate either from a heat-labile component shared with the lived pathogen or from a rapidly expressed cellular or bacterial factor acting on the transcription rate of the tested genes (Figure 1a). As a control, the mRNA expression of the bacterial PAD was only detected in HGEC infected with *P. gingivalis*, indicating that the pathogen might penetrate HGEC to be active (Figure 1a).

### Effect of LPS-Pg on mRNA expressions of SOCS-3, IRF-1, EGF, EGFR, and STAT-3

Among all the components and virulence factors expressed by *P. gingivalis*, the study tested whether its purified LPS, a potent pro-inflammatory mediator, leads to the similar downregulated effects observed with the live pathogen. HGEC were stimulated by 0.5 µg/mL and 1 µg/mL of purified LPS for 2 and 24 h, and mRNA expression levels of the tested genes were determined by RT-qPCR. During the time-course activation of HGEC, and compared to the corresponding non-activated control cells, only the expression level of the mRNA encoding for IRF-1 was increased after 2 h of activation (3.3-fold). This increase was dose-dependent, since the use of 0.5 µg/mL of purified LPS also led to a significant increase, although this was reduced compared to the LPS-*Pg* dose of 1 µg/mL (1.5-fold; Figure 1b). After 24 h of activation, mRNA expression of IRF-1 reached the maximum (5.5-fold), while significant increases in expression levels of mRNAs encoding for SOCS-3 (3.5-fold) and EGFR (2.5-fold) started to be measured. These effects were also dose-dependent, as the use of 0.5 µg/mL of purified LPS-*Pg* led to significant but reduced increases compared to the 1 µg/mL dose (1.7-fold for SOCS-3 and 1.5-fold for EGFR). LPS-*Pg* doses <0.5 µg/mL did not significantly modify the expression levels of all tested mRNA (data not shown). No variation in expression levels of mRNAs encoding for EGF and STAT-3 were measured at any point during the time-course activation of HGEC and with any dose of LPS-*Pg* used (Figure 1b).

### HGEC infection with *P. gingivalis* inhibited the protein expression of SOCS-3, IRF-1, and EGFR but not STAT-3

**Figure 2.**
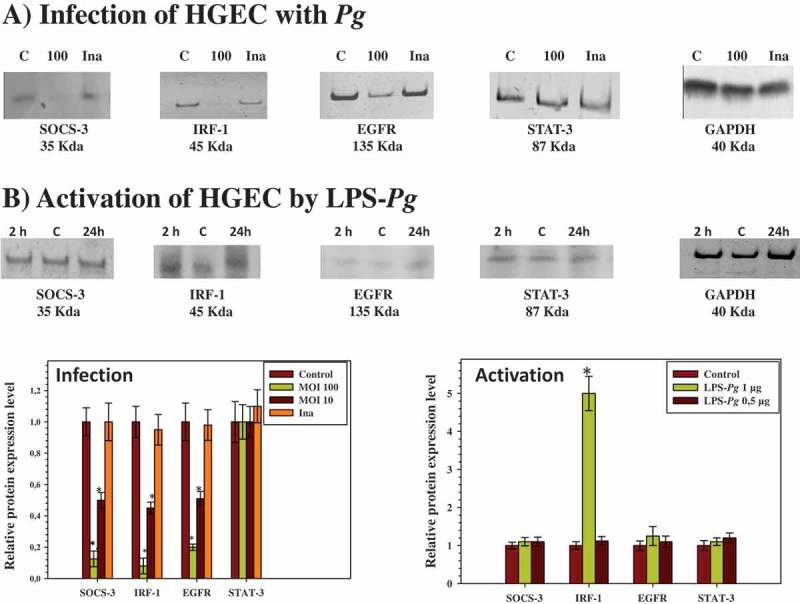
Modulation of the expression of SOCS-3, IRF-1, EGFR, and STAT-3 proteins in HGEC infected with *P. gingivalis* and activated by LPS-*Pg*. (a) HGEC were infected for 2 h with *P. gingivalis* at a MOI of 100 and with heat-killed *P. gingivalis* (Ina). Uninfected cells served as control (C). (b) HGEC were activated 2 and 24 h by 1 µg/mL of purified LPS-*Pg*. Non-activated cells served as controls (C). In both experiments, cellular extracts were prepared and analyzed by immunoblotting with antibodies to SOCS-3, IRF-1, EGFR, and STAT-3. No detection was obtained using an antibody raised against EGF (not shown). An antibody to GAPDH was used as an internal control to verify equal loading of total proteins in all wells. Histograms indicated the relative protein expression level during infection and activation. Levels were determined by pixel intensity of a protein band normalized to the intensity of the internal control GAPDH within the same assay. Differences (*) between a given ratio and the one obtained with control cells were analyzed with Student’s *t*-test (*p* < 0.0005).

Next, the study evaluated whether the expression of each protein corroborated with the variation of its specific mRNA measured by RT-qPCR during infection of HGEC. Cells were infected for 2 h at different MOIs with *P. gingivalis* and heat-killed *P. gingivalis*. Cellular extracts were prepared for detection by Western blotting of SOCS-3, IRF-1, EGF, EGFR, and nuclear extracts prepared for the detection of STAT-3 proteins. Compared to the corresponding protein level measured in uninfected cellular extracts, the infection performed with *P. gingivalis* at a MOI of 100 significantly reduced the expression level of SOCS-3 (5-fold), IRF-1 (5-fold), and EGFR (6-fold), but not STAT-3, in the nuclear extracts (Figure 2a). These decreases were not significantly visualized when the infection was performed with the heat-killed *P. gingivalis* (Figure 2a) and with live *P. gingivalis* at a MOI of 10 (data not shown). No band corresponding to the EGF protein was detected, reflecting the low mRNA expression level measured previously by RT-qPCR and thus explaining the need to supplement the culture medium of HGEC with rEGF to stimulate cell growth.

### Activation of HGEC by LPS-Pg stimulated IRF-1 protein expression

When HGEC were activated by 0.5 µg/mL and 1 µg/ml of purified LPS-*Pg* for 2 and 24 h, a significant increase was observed only in the expression of the IRF-1 protein at both time points (Figure 2b) and solely with the dose of 1 µg/mL. As normalized to the non-activated control HGEC, this increase was calculated to be threefold at each of the two points of the time-course activation of the cells. No significant variation of the intensity of the IRF-1 band was observed using a LPS-*Pg* dose of 0.5 µg/mL (data not shown). Bands corresponding to SOCS-3, EGFR, and STAT-3 did not significantly vary during the experiments whatever the LPS-*Pg* dose employed (data not shown). No band for the EGF protein was detected at any time of the activation and with any LPS-*Pg* dose used (data not shown).

### Tyrosine-phosphorylation of STAT-3 increased during infection of HGEC with *P. gingivalis* but decreased during activation by LPS-Pg

Previous studies demonstrated the role of p-STAT-3 in transmitting signals within nuclei as a result of EGF signaling [27]. Therefore, to corroborate the implication of STAT-3, the tyrosine-phosphorylation level of this transcription factor at Tyrosine^705^ was investigated after infection and activation of HGEC. As Tyr-phosphorylated-STAT-3 (p-STAT-3) is translocated within the nucleus of cells, nuclei extracts were prepared from infected and activated HGEC to visualize any change in the phosphorylated status of STAT-3 by Western blots.

**Figure 3.**
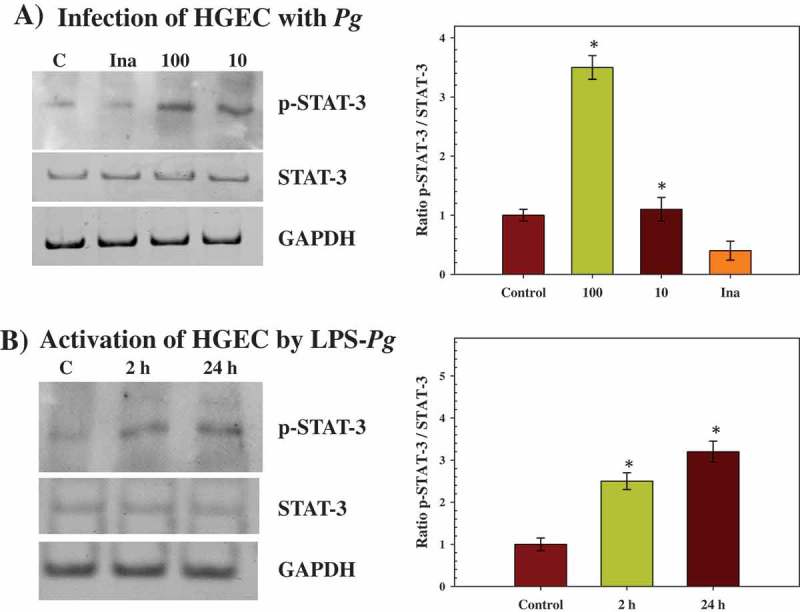
Effect of infection with *P. gingivalis* and activation by LPS-*Pg* of HGEC on the phosphorylation status of STAT-3 protein (p-STAT-3). (a) HGEC were infected for 2 h with *P. gingivalis* at a MOI of 100 and with heat-killed *P. gingivalis* (Ina). Uninfected cells served as control (C). (b) HGEC were activated for 2 and 24 h by 1 µg/mL of purified LPS-*Pg*. Non-activated cells served as controls (C). In both experiments, nuclear extracts were prepared and analyzed by immunoblotting with anti-phospho-STAT-3 (Tyr^705^) antibody to detect p-STAT-3 and with anti-STAT-3 antibody to reveal total STAT-3 protein. The antibody for GAPDH was used as an internal control to verify equal loading of proteins in all wells. Each band was quantified by densitomery and the band intensity obtained with anti-phospho-STAT-3 (Tyr^705^) antibody in an assay was expressed relative to the intensity of the band obtained with the antibody for total STAT-3 antigen in the same assay. Histograms showed changes of the ratio p-STAT-3/STAT-3 during infection and activation of HGEC. Differences (*) between a given ratio and the one obtained with control cells were analyzed with Student’s *t*-test (*p* < 0.0005). All experiments were repeated three times, and the results are expressed as mean ± the standard deviation.

A significant increase of p-STAT-3 (3.5-fold) was measured during infection of HGEC performed with *P. gingivalis* at a MOI of 100, while no difference was observed with an infection performed at a MOI of 10 (Figure 3a). However, a significant decrease (2.5-fold) was observed when the infection was performed with heat-killed *P. gingivalis* (Figure 3a). During the time-course activation of HGEC by LPS-*Pg*, significant increases of p-STAT-3 were measured at 2 h (2.5-fold) and at 24 h (3.2-fold) (Figure 3b).

## Discussion

During periodontitis, HGEC are the initial host cells encountered by organisms such as *P*. *gingivalis* that colonize the epithelium pocket. Among the cell-derived factors whose expression is probably modified in response to bacterial invasion, EGF and EGFR contribute actively to the normal periodontal tissue turnover and to periodontal tissue repair and regeneration during chronic inflammatory periodontal diseases [13]. However, until now, little was known about the regulation of the EGF pathway during periodontitis, notably how *P. gingivalis* and one of its virulent factors (i.e. LPS) could modify the signaling cascade initiated by EGF. The data obtained with HGEC clearly indicated that *P. gingivalis* can modify the expression of various proteins acting on the EGF signaling pathway, but in a way that depends on how the periodontopathogen acts, either as a live bacteria able to invade the target cells or solely through its potent pro-inflammatory LPS. In the present model of HGEC infected with *P. gingivalis* and activated by LPS-*Pg*, the infection led to a dose-dependent inhibition of SOCS-3, IRF-1, and EGFR expression, both at the transcriptional and at the protein level, while the cell activation by LPS-*Pg* increased with time for these three tested gene expressions.

In HGEC infected with *P. gingivalis*, inhibition of SOCS-3 expression may represent a way by which the bacterium contributes to chronic inflammation associated with periodontitis. An inhibition of SOCS-3 expression during infection has been associated with an increased severity of inflammation in patients with Down syndrome (DS) and periodontitis in comparison to either euploid individuals affected by periodontal diseases or periodontally healthy euploid individuals [28]. The molecular mechanism underlying the SOCS-3 inhibition during infection probably occurred through the induction of mRNA-203, a small single-stranded noncoding RNA that can suppress SOCS-3 expression at both mRNA and protein levels by binding to the 3ʹ non-translated region of SOCS-3 mRNA. Such a molecular hypothesis has been described in a similar model of gingival epithelial cells infected with *P. gingivalis* [29]. A second molecular hypothesis could involve one or different bacterial component(s) able to influence the transcription or the translation of the mRNA encoding SOCS-3. This latter hypothesis was endorsed by the fact that inhibition of SOCS-3 was completely removed when whole bacteria were previously heat killed, suggesting that such component(s) are either transported by the live bacterium or immediately synthetized within the cells during early stages of bacterial invasion and proliferation. Whatever the molecular mechanism explaining the inhibition of SOCS-3 expression during infection, it was probably not implicated during activation of HGEC by purified LPS-*Pg*. Indeed, after 24 h of cell activation, a significant increase was observed of the expression level of the mRNA encoding SOCS-3 but not of the protein. The molecular mechanism that might explain the discrepancy between infection and activation of HGEC for the expression of SOCS-3 remains to be elucidated, but this discrepancy certainly reflected the activation of a different signaling pathway in host immune/inflammatory responses to LPS-*Pg*, as demonstrated in human monocytes infected with live *P. gingivalis* and stimulated by its purified LPS or its major fimbrial protein, fimbrillin (FimA) [30].

IRF-1 is also a protein differentially affected by the presence of either the live *P. gingivalis* or its purified LPS. The downregulation of the expression of mRNA encoding IRF-1 (12-fold) was comparable to the one observed elsewhere (8-fold) in gingival epithelial cells infected with *P. gingivalis* [25]. A significant reduction of the IRF-1 mRNA expression was also associated with DS patients compared to euploid individuals affected by periodontitis [31]. As IRF-1 regulated a diversity of cellular responses, the downregulation observed after infection of HGEC probably contributed to targeting genes possessing an interferon-stimulated response element within the promoter sequence, as demonstrated by the inhibition of cytokines IP-10, ITAC, and Mig expressions in epithelial cells [25]. As observed with SOCS-3, the experiments performed in HGEC activated by LPS-*Pg* led to an increase in the expression level of mRNA encoding IRF-1, with a maximum level observed after 24 h of activation (Figure 2b). LPS is known to stimulate several signaling pathways, including nuclear factor-κB and the mitogen-activated protein kinase [32]. Therefore, in the cellular model, LPS-*Pg* activation of IRF-1 expression was probably an essential step to mediate the expression of various inflammatory factors and to impact important cellular processes. Likewise, IRF-1 has been shown to be an essential transcription factor for LPS-induced iNOS expression because LPS failed to induce iNOS expression in IRF-1-deficient macrophages [33]. Moreover, maximal expression of the IL-1β gene was observed in murine cells that co-expressed IRF-1, IRF-2, and IRF-4 [34]. In addition, the activation of the lymphotoxin-beta receptor induced expression of IRF-1, one of the major transcription factors of IL-8 gene [35]. Other data support evidence that IRF-1 activation is also necessary to inhibit autophagy [36].

We also examined the expression of EGF and its receptor EGFR that were profoundly modified during periodontal disease. Concerning EGF, a very low level of expression of both the mRNA and the protein were found, with a consequent need to supplement the cell culture medium with purified recombinant EGF to stimulate cell proliferation. Additionally, no significant variation of EGF expression was observed during HGEC infection and activation. However, the data showed that the expression of mRNA encoding EGFR was significantly downregulated as a result of the infection with *P. gingivalis*. This result was different from other data indicating that inflammation of human gingiva, associated with the development of periodontitis, leads to an enhanced expression of EGFR. Indeed, in healthy gingiva, using immunohistochemistry, EGFR expression was limited to the gingival epithelium, but during the development of periodontal diseases, a significant increase was only observed in the periodontal ligament [13]. It is assumed that depending on the inflammation status of the gingiva, live bacteria act differently on the cell types with which they come in contact. In the cellular model, during HGEC activation by LPS-*Pg*, the expression of mRNA encoding EGFR was upregulated but only after 24 h, while the protein level did not vary significantly. The difference in the expression of EGFR during infection and activation of HGEC was a discrepancy that has also been observed in human monocytes, reflecting the fact that live bacteria signaled mostly through TLR-2 and TLR-4, while purified LPS-*Pg* acted through TLR-2 [37].

Such a discrepancy was not observed in experiments performed to highlight the potential role of STAT-3. Indeed, HGEC infected with *P. gingivalis* and activated by LPS-*Pg* significantly increased (3.5- and 3.2-fold, respectively) the percentage of STAT-3 phosphorylation at tyrosine^705^, while the infection with heat-killed *P. gingivalis* leads to a significant decrease (2.5-fold). The increase in STAT-3 phosphorylation after *P. gingivalis* infection and LPS-*Pg* activation has already been observed in gingival epithelial cells and other types of cells [30,38]. During infection of HGEC, the increase was not dependent on the modulation of STAT-3 mRNA expression levels, since no difference was measured when compared to the uninfected control cells, suggesting that STAT-3 activation is a process that occurs very rapidly within cells. Moreover, it has been shown that STAT-3 activation may be a process that occurs in a SOCS-3-independent mechanism [22,39]. The present results supported this hypothesis, since SOCS-3 expression was significantly reduced, while STAT-3 phosphorylation increased in gingival epithelial cells infected with *P. gingivalis*. This SOCS-3-independent mechanism of STAT-3 activation was probably not involved when LPS-*Pg* was used. As the expression of SOCS-3 protein did not significantly vary during LPS-*Pg* activation, it was assumed that the increase of STAT-3 phosphorylation level constituted a SOCS-3-dependent process. Whatever the SOCS-3 mechanism was that explains the increasing phosphorylation of STAT-3, the present data are in agreement with those showing an activation of this transcriptional factor in response to the LPS-*Pg* [40].

The present experiments highlight how *P. gingivalis* and its LPS modulate multiple pathways in epithelial cells to achieve infection, differential cytokine expression, and host responses. These experiments contributed to the understanding of the complex networks of interactions found between *P. gingivalis* and its purified LPS in the modulation of the expression of various molecules (SOCS-3, IRF-1, EGF-R, and STAT-3) influencing the EGF signaling in gingival epithelial cells. In periodontitis, understanding the molecular basis of host responses might help to prevent infection and minimize the tissue damage that results from an aggressive host response.
